# Obesity, low-grade inflammation, and inflammatory response to immune challenge modulate willingness to expend effort for reward

**DOI:** 10.1016/j.bbi.2026.106548

**Published:** 2026-03-13

**Authors:** Iris Ka-Yi Chat, Lina S Hansson, Mats Lekander, Charlotta Jacobsen, Sven Benson, Johannes Hebebrand, Vera Bender, Analena Handke, Till Hasenberg, Michael Treadway, Harald Engler, Robert Dantzer, Manfred Schedlowski, Julie Lasselin

**Affiliations:** a Department of Psychology, University of California, Los Angeles, USA; b University of Pittsburgh Medical Center, USA; c Department of Psychology, Stockholm University, Sweden; d Department of Clinical Neuroscience, Division of Psychology, Karolinska Institutet, Sweden; e Osher Center for Integrative Health, Department of Clinical Neuroscience, Karolinska Institutet, Sweden; f Institute of Medical Psychology and Behavioral Immunobiology, Center for Translational Neuro- and Behavioral Sciences, University Hospital Essen, University of Duisburg, Essen, Germany; g Department of Child and Adolescent Psychiatry, Psychosomatics and Psychotherapy, University of Duisburg, Essen, Germany; h Clinic for Obesity and Metabolic Surgery, Sana Klinikum Remscheid GmbH, Remscheid, Germany; i Emory University, Atlanta, USA; j University of Texas MD Anderson Cancer Center, Houston, USA; k Department of Biomedical and Clinical Sciences, Linkӧping University, Sweden

**Keywords:** Lipopolysaccharide, Cytokines, C-reactive protein, Inflammation, Motivation, Reward, Anhedonia, Obesity

## Abstract

**Background:**

Inflammation is increasingly considered a vulnerability factor for deficits in reward motivation. It remains unclear if such motivational changes differ between acute and chronic low-grade inflammation. Given obesity’s link to chronic low-grade inflammation, the present study *a priori* tested the effects of obesity and low-grade inflammation (elevated CRP concentrations), as well as acute inflammation (experimental immune challenge using lipopolysaccharide), on motivation for a monetary reward. Moreover, the wear-and-tear induced by chronic inflammation could amplify motivational changes induced by immune challenge. Thus, we also tested the *a priori* hypotheses that acute inflammation is associated with more strongly altered reward motivation for individuals with higher low-grade inflammation, and for obese than normal-weight individuals.

**Methods:**

Obese (n = 14) and normal-weight (n = 21) young adults, all without current or past history of medical or psychiatric conditions, were intravenously injected with a bacterial endotoxin (lipopolysaccharide, LPS [0.8 ng/kg body weight]) to trigger an acute inflammatory response, and placebo (saline) in a counterbalanced, randomized, crossover design. The Effort Expenditure for Reward Task (EEfRT) was administered 2 h following each injection to assess reward motivation. Blood was sampled pre- and post-injection to quantify concentrations of inflammatory proteins (interleukin [IL]-6 and C-reactive protein [CRP]).

**Results:**

Obesity and higher low-grade inflammation (baseline CRP) were associated with overall reduced motivation to expend effort for reward in the placebo condition. Interactions with reward magnitude were observed, so that this effect occurred at smaller reward values, but not when reward values increased. However, these interaction effects were not significant in *a posteriori* sensitivity analyses. LPS-induced inflammatory responses (IL-6 concentrations during EEfRT) were associated with a weaker influence of reward magnitude on increasing effort. Unexpectedly, we did not detect the amplification effect of obesity and its associated low-grade inflammation on the ability of lipopolysaccharide to modulate reward motivation.

**Conclusions:**

Lipopolysaccharide-induced and low-grade inflammation may alter how effort is allocated towards reward: low-grade inflammation reduced the willingness to expend effort, while acute inflammation dampened reward sensitivity. This study offers a nuanced understanding of inflammatory processes underlying alterations in reward motivation.

## 1. Introduction

Alterations in reward motivation are implicated in a broad range of neuropsychiatric disorders and symptoms (Pizzagalli, 2022; Treadway & Zald, 2011). These motivational deficits as well as other aspects of anhedonia are among the most difficult to treat (Pizzagalli, 2022). Identifying factors that modulate reward motivation could offer insight into ways of addressing the public health concerns from a transdiagnostic perspective.

Over the past few decades, inflammation has garnered attention in psychopathology research (Bower & Kuhlman, 2023; Dantzer et al., 2008; Maes, 1995; Miller & Raison, 2016; Nusslock et al., 2024). This rapidly expanding literature originates from conceptual frameworks which propose that inflammation can modulate brain function, leading to a constellation of behavioral changes known as sickness behavior and resembling symptoms observed across various neuropsychiatric disorders (Dantzer & Kelley, 2007; Lasselin et al., 2021). From an evolutionary perspective, behavioral changes induced by inflammation are believed to serve to reorganize metabolic resources towards survival priorities during sickness. These changes are typically adaptive responses. However, chronic inflammation persisting in the absence of an immune challenge or disproportionate inflammatory responses can lead to maladaptive outcomes, including neuropsychiatric symptoms (Dantzer et al., 2008; Vichaya & Dantzer, 2018).

Behavioral adjustments during sickness represent a strategic reallocation of effort in response to physiological demands, rather than a general decrease in effort for all reward incentives (Vichaya & Dantzer, 2018). This is achieved by suspending non-essential activities while optimizing the allocation of effort expenditure. For instance, studies have shown that during sickness, individuals prefer activities that require less physical or cognitive effort (e.g., resting) or make choice that minimizes costs and optimizes benefits (e.g. obtaining care from others or obtaining comforting rewards), which are behaviors conducive to recovery (Boyle et al., 2019; De Marco et al., 2023; Draper et al., 2018; Eisenberger et al., 2010, 2017; Hansson et al., 2024; Harrison et al., 2016; Lambregts et al., 2023; Lasselin et al., 2017; Vichaya & Dantzer, 2018). Thus, inflammation may dynamically regulate motivational behaviors based on the contexts, prioritizing activities that support immune function during sickness (Lasselin et al., 2017).

The implications become more complex when considering individuals with pre-existing chronic low-grade inflammation, which indicates dysregulation in the immune system. While acute LPS-induced increases in inflammatory proteins are linked to sickness responses and behavioral changes (Dooley et al., 2018), individuals with pre-existing chronic inflammation may exhibit heightened susceptibility to these effects. During chronic inflammatory states, immune-to-brain signaling pathways may be more permissive, such that a given inflammatory signal is more likely to alter behavior. Indeed, chronic inflammatory states and relevant conditions, such as obesity (Gregor & Hotamisligil, 2011; Lasselin et al., 2014), have both been associated with elevated vulnerability to neuropsychiatric symptoms (Dantzer et al., 2008; Stunkard et al., 2003). Also consistent with this perspective, animal studies demonstrate exaggerated sickness responses and depressive-like behaviors following LPS administration in obese rodents relative to lean controls (Aguliar-Valles et al., 2014; Lawrence et al., 2012; Pohl et al., 2014). Furthermore, prior work indicates that chronic inflammation may be associated with a lower threshold at which a subsequent immune challenge influences brain and behavior, via alterations in immune-brain interface, including increased permeability in the blood–brain barrier (Huang et al., 2021). These effects could extend to the dopamine-driven mesocorticolimbic circuit that governs motivational behavior (Treadway et al., 2019), where individuals with chronic low-grade inflammation show more significant motivational changes in response to acute immune challenges compared to those with lower levels of inflammation.

However, there are several methodological challenges to test such amplification effect. Systemic inflammation and its resulting behavioral changes may not necessarily represent a pathological process but an adaptive response when fighting infection, injury, or sickness, making it difficult to operationalize, measure, and interpret the amplification effects. Moreover, ensuring sufficient variations in levels of chronic low-grade inflammation, especially in healthy individuals, is challenging, and inducing chronic low-grade inflammation experimentally in healthy individuals is unethical. To capture a chronic low-grade inflammatory state interacting with responses to immune challenge, a study design needs to mitigate these methodological limitations, for instance by using a recruitment method that would allow for sufficient variations in chronic low-grade inflammation and involve illness-free participants. However, to our knowledge, such experimental endotoxemia research is sparse (Lasselin et al., 2020).

Recruitment based on obesity status provides a way to capture variations in levels of chronic, low-grade inflammation. Obesity often precedes multiple psychiatric and physical health conditions that have an immune basis (Kanneganti & Dixit, 2012) and is characterized by sustained immune activation driven by trafficking of macrophages into the adipose tissue and alterations in gut permeability, which results in elevated circulating inflammatory proteins (Cox et al., 2015; Gregor & Hotamisligil, 2011; Park et al., 2005). Among these inflammatory proteins, baseline C-reactive protein (CRP) reflects cumulative inflammatory activity over longer timescales and is commonly used as an index of chronic, low-grade inflammation, particularly in the absence of acute immune challenge (Banks et al., 1995; Kemna et al., 2005; Kushner et al., 2006) – and research has documented association between baseline CRP and reward motivation (Felger et al., 2016). In contrast, cytokines, particularly interleukin-6 (IL-6), respond rapidly to immune challenge, peaking within hours following LPS administration, and thus index acute inflammatory reactivity (Kemna et al., 2005). Such acute IL-6 responses following LPS have been consistently observed across experimental studies, and emerging evidence suggests their relevance for altered reward motivation (Boyle et al., 2025).

Although both acute and chronic inflammatory processes have been independently examined in relation to reward motivation (Boyle et al., 2025; Dooley et al., 2018; Felger & Treadway, 2017; Savitz et al., 2025), these distinct temporal features may have different implications for reward motivation. Integrated evolutionary and reinforcement learning frameworks suggest that acute inflammatory responses, indexed by IL-6, are thought to elicit transient behavioral adjustments that promote adaptation to imminent survival demands, whereas chronic low-grade inflammation, indexed by baseline CRP, is more likely to reflect dysregulated immune-brain signaling associated with persistent alterations in reward motivation (Dantzer, 2023; Lacourt et al., 2018). Examining both baseline CRP and LPS-induced IL-6 within the same sample therefore provides a unique opportunity to assess whether acute and chronic inflammatory activity alter reward motivation in overlapping or distinct ways and allow for examining whether chronic inflammation moderates the effects of acute inflammatory reactivity on reward motivation.

### 1.1. Current study

The primary aim of the current study was to examine motivation for a monetary reward following an immune challenge among obese and normal-weight individuals. We first assessed the effect of obesity versus normal-weight (aim 1a), as well as the impact of baseline chronic inflammation (elevated CRP concentrations) (aim 1b), commonly observed in obesity, on reward motivation; and the effect of the immune challenge (i.e., intravenous injection of lipopolysaccharide, LPS) versus placebo (saline) (aim 2a) and the strength of the inflammatory response (aim 2b) on reward motivation. Then, drawing on converging evidence indicating that individual differences in IL-6 responses to acute immune challenge were associated with altered reward motivation, (Boyle et al., 2025; Lasselin et al., 2017), we investigated whether the inflammatory responses to an immune challenge represented by systemic lipopolysaccharide would be associated with a stronger decreased willingness to expend effort for reward in obese than in normal-weight individuals (aim 3). Given our rationale that chronic low-grade inflammation may amplify the effect of immune challenge on motivational reward processing, we also assessed whether a higher baseline low-grade inflammation would amplify this association (aim 4).

To address these aims, the current study employed a randomized double-blind, placebo-controlled, crossover design. Participants completed the Effort Expenditure for Rewards Task (EEfRT; Treadway et al. [2009]), a computerized task to assess willingness to expend physical effort (button presses) for monetary reward following the administration of lipopolysaccharide versus placebo, with a 2-week washout between treatments. Although the parent study from which current study derives (Lasselin et al., 2020) did not detect differences in inflammatory responses or behavioral changes following LPS administration between obese and normal-weight individuals, the report focused on subjective behavioral changes and not reward-related motivation. Similarly, prior work examining obesity status and reward motivation has found reduced reward motivation among obese individuals; however, inflammatory markers were not assessed (Mata et al., 2017). Thus, it remains unclear whether previously observed differences in reward motivation were attributable to low-grade inflammation underlying obesity or obesity itself more generally, and whether motivational changes in response to immune challenge are amplified in high levels of low-grade inflammation or in obesity.

## 2. Methods and Materials

### 2.1. Participants

Forty adults (55% female; *M*_age_ = 26) were enrolled based on their obesity status as indexed by body mass index (BMI [kg/m^2^]), Obesity (OB; *n* = 15, *Range*_BMI_ > 30; M_BMI_ = 38.0) vs. Normal Weight (NW; *n* = 25, Range_BMI_ = 18.5–25.0; *M*_BMI_ = 22.7). Exclusion criteria included tobacco and excessive alcohol consumption, diagnosis of somatic or neuropsychiatric disorders, abnormalities in electrocardiogram or blood analysis (e.g., indicating metabolic dysfunction), current medication use, and infection within the past two weeks. As in Lasselin et al. (2020), 3 of the 40 participants were excluded from the analyses because they were outliers for baseline concentrations of inflammatory markers (i.e., 5 SD above the mean) which indicated possible infections (*n* = 2 from the NW group), and because of a possible depression indicated by high scores on the Beck Depression Inventory (*n* = 1 from the OB group). In the current study, additional participants were excluded from the analyses because of factors that might affect task experience: EEfRT completed on the desk instead of a bed (*n* = 1 from the NW group), ambiguous-handedness (n = 1 from the NW group), and pain in the arm due to catheter (*n* = 1 from the OB group during Placebo Condition).Thus, the final sample in the current study consisted of 35 participants, 21 of whom were of normal weight and 14 who were obese (of whom 13 completed the Placebo condition).

### 2.2. Study design

The current study derived from a study conducted at University Hospital Essen, Germany (Lasselin et al., 2020). It employed a randomized double-blind, placebo-controlled, counterbalanced, crossover design, with a 1–2 week washout between treatments, where participants were injected with lipopolysaccharide (LPS) as immune challenge at one occasion, and saline as placebo at the other occasion (in a randomized order), at hour 0 (see Lasselin et al. [2020] for details). The dose of LPS was a function of the participant’s body weight (see 2.3). Blood draws and self-rated questionnaires were administered every hour. At the peak of the inflammatory response, i.e., 2–3 h post-injection, a computerized reward task was administered as an ancillary component (see 2.5), added prior to data collection of the parent study to assess reward-based motivation during acute inflammation, which was not a focus of the original study (Lasselin et al., 2020). Medical signs were measured every 30 mins. The study was conducted by medical students under the supervision of a medical doctor.

The study was approved by the Institutional Ethics Review Board of the Medical Faculty of the University of Duisburg-Essen (permit number: 15–6503-BO) and from the Swedish Ethical Review Authority (to process study data in Sweden, 2022–00664–01). All subjects enrolled provided signed informed consent after receiving a written and oral description of the study, and received financial compensation (310–360€) for their participation in the study.

### 2.3. LPS vs. Placebo administration

The experimental treatments consisted of an intravenous injection of either saline (NaCl 0.9%, sterile, pyrogen-free physiological saline solution B Braun Melsungen, Melsungen, Germany) in the control (placebo) condition or LPS (Endotoxin Reference Standard, *Escherichia coli* O113:*H*10, CAT number: 1235503, lot H0K354, United States Pharmacopeia, Rockville, MD, USA). Normal-weight participants were injected with 0.8 ng/kg body weight of LPS. The dosage for obese participants was adapted to their estimated blood volume because the ratio of blood volume to body weight is lower in obese individuals (Lemmens et al., 2006). This resulted in doses of 0.51 to 0.68 ng/kg body weight for obese participants. Lasselin et al. (2020) showed in their analysis of the same data that the sickness behavior in the obese and normal-weight groups was very similar after this differential treatment.

### 2.4. Inflammatory markers

Plasma concentrations of the inflammatory cytokines, IL-6 and TNF-α were assessed in duplicate by using a magnetic bead-based multiplex assay (Human Mag Luminex Performance Assay, LHSCM000, LHSCM206, LHSCM208, LHSCM210, R&D Systems, MN, USA) on a Luminex 200 System (software xPONENT 3.1, Luminex corporation, Austin, Texas) according to the manufacturer’s protocol. The minimum detectable dose (MDD) of the assay was 0.28 pg/mL for IL-6 and 0.58 pg/mL for TNF-α. Nineteen (3.4%) and four (0.7%) samples at baseline and post-placebo injection were below the MDD levels of IL-6 and TNF-α, respectively, and MDD values were then used for statistical analyses. Of note, IL-6 and TNF-α were all detectable in the blood samples 1–6 h after the LPS injection. Plasma concentrations of CRP were measured using an enzyme-linked immunosorbent assay (ELISA; Human CRP Quantikine ELISA, DCRP00, R&D Systems, Minneapolis, MN, USA) using duplicates and according to the manufacturer’s protocol (but adjusting the sample dilution to 1:1000 instead of 1:100). As CRP concentrations after LPS administration started rising at 6 h after LPS injection (Engler et al., 2017), CRP concentrations were measured at baseline, 6 h and 24 h after the injection. Only baseline CRP concentrations are used in the current study. The assay’s sensitivity was 0.01 mg/L, and concentrations lower than 0.01 mg/L in three (1.3%) samples were replaced by 0.01 mg/L.

Plasma concentrations of IL-6 (rather than TNF-α or CRP) at the time of EEfRT (average at 2–3 h post-LPS) were chosen *a priori* as the primary index of the inflammatory response to LPS. This choice was consistent with prior work on LPS-induced IL-6 and alterations in reward motivation (Boyle et al., 2025; Lasselin et al., 2017). Furthermore, peak IL-6 concentrations at this window represent the largest effect of LPS administration and are thought to best reflect maximum immune signaling to the brain (Fig. 2 in Lasselin et al., 2020). In addition to IL-6, exploratory analyses using TNF-α were conducted (average at 2–3 h post-LPS and at 1–2 h post-LPS given that TNF-α concentrations peak earlier, Lasselin et al., 2020); and are reported in Supplementary Table S3.

### 2.5. Effort Expenditure for rewards task (EEfRT)

**Fig. 1.**
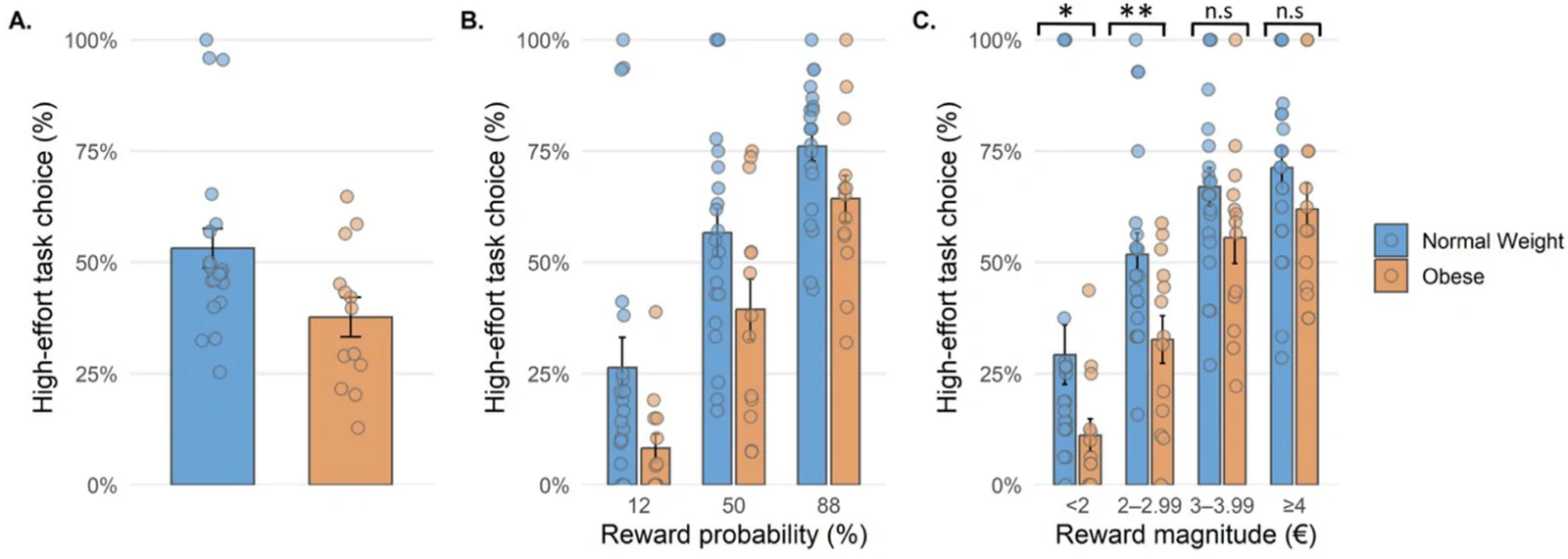
Effect of group (obese vs. normal weight) on high-effort task choice (%) across all trials (A), by reward probability (B), and by reward magnitude (C) in the placebo condition. The reward magnitude (1.24–4.21 €) was divided into four categories (<2€, 2–2.99€, 3–3.99€, and ≥ 4€) for exploratory post-hoc analyses. Note that the interaction obesity*reward magnitude was not significant in sensitivity analyses removing three individuals with extremely high percentage of hard-task choices (>95%) and should thus be cautiously interpreted. Individual data points represent proportion of high-effort task choices for each participant and has been jittered. Error bars represent the standard error of the mean. Asterisks indicate significant results. *p < 0.05, **p < 0.01, n.s. = non-significant.

The EEfRT developed by Treadway et al. (2009) was conducted between 2 h and 3 h after LPS or placebo administration on a laptop placed on a bedside table. The EEfRT takes 20 min and includes as many trials as can be completed within that timeframe (see Fig. 1 for illustration). The participants were told that in each trial they must choose between a low-effort/low-reward and a high-effort/high-reward alternative; in the following, these are referred to as “easy task” and “hard task”, respectively, for better readability. The easy task requires 30 button presses on a computer keyboard in 7 s with the index finger of the dominant hand and can be rewarded with 1 €. The hard task requires 100 button presses in 21 s with the little finger of the non-dominant hand and can be rewarded with 1.24–4.21 €. Two factors vary between trials: the probability that the participant receives the reward after successfully completing the easy or hard task (12%, 50%, 88%), and the reward magnitude for the hard task (1.24€ to 4.21€). Participants had four practice trials before starting the task. To ensure that the participants remained motivated throughout this task, they were informed that only four trials would be randomly selected at the end of the EEfRT. Based on rewards won during the task across the two study days, participants received an additional bonus of 10–40€, on top of a base compensation of 300–320€ for participation in the larger study.

The outcome variable of the EEfRT is whether the participant chooses the hard task over the easy task in a given trial (hard task choice). The proportion of hard task choices over easy task choices reflects incentive motivation. Incentive motivation can also vary depending on the reward probability. The effect of the reward magnitude on the hard task choice reflects reward sensitivity. To ensure that the hard task choices in the EEfRT primarily reflect motivated behavior and not merely the physical capability of performing the hard task, decision time to choose between the two alternatives and the task performance (i.e., rate of successful trials and time to complete the task) were examined. Effort as a function of reward magnitude and probability were included in the models as continuous variables on their original scale. Trials (120 of 4467; 2.7%) were excluded on a trial-by-trial basis using listwise deletion within the GEE framework. Trials were removed when participants did not make a task choice within the five-seconds window (31 trials, resulting in forced choice), due to technical problem (2 trials), or when participants made a choice implausibly fast (i.e., in less than 500 ms; 87 trials).

### 2.6. Statistical analyses

Demographics data between the obese and normal-weight groups were assessed using t-tests and χ^2^ test. Overall performance in the EEfRT, that is, reaction time to choice and completion time, between the obese and normal-weight groups in the LPS and placebo conditions were assessed using linear mixed models with obesity, condition, and obesity*condition as fixed effects; session number and EEfRT trial as repeated variable with AR1 covariance structure; and a random intercept. All residuals were normally distributed as indicated by skewness < |2| and kurtosis ≤ |5|. Because successful rate was high and not normally distributed, the proportion of individuals with 100% successful rate was compared between the two groups and conditions using χ^2^ test.

#### Aim1:

A generalized estimating equation (GEE) was first used to assess the effect of obesity versus normal-weight status (aim 1a), as well as the interaction effects of obesity with reward magnitude and reward probability, on the proportion of high-effort task choices in the placebo condition. Next, to assess the effect of chronic inflammation on EEfRT performance (aim 1b), a GEE model was used testing the effect of baseline CRP, as well as the interaction effects of baseline CRP with reward magnitude and reward probability, on high-effort task choices across all participants in the placebo condition.

#### Aim 2:

Two similarly structured GEE models were conducted with either condition (LPS vs. placebo) (aim 2a) or IL-6 concentration (aim 2b) as the main effect, to test the impact of acute inflammation on high-effort task choices. Exploratory analyses were conducted to assess the effect of TNF-α concentration.

#### Aims 3 and 4:

The interaction effect between obesity and acute inflammation, as well as between baseline CRP and acute inflammation, was then tested. IL-6 concentrations and the interactions with obesity/baseline CRP were added to the models tested in Aim 1. Note that the models with condition (LPS/Placebo) were not tested because they were not found to be significant in aim 2.

An exchangeable variance structure was used in all GEE models, and the models were adjusted for EEfRT trial, session number, sex, and age. However, sex had to be removed as covariate for some models that did not converge: original model with obesity in 3.1, original models with LPS and IL-6 in 3.2. When an interaction with probability or reward magnitude was found, exploratory post-hoc analyses were conducted, analyzing the effect of at each probability level (12%, 50%, 88%) and at reward magnitude categories (<2€; 2.2.99€, 3–3.99€, ≥4€).

Three normal-weight individuals had an extremely high percentage of hard-task choices (>95%) in both the LPS and the placebo conditions. Therefore, we performed sensitivity analyses by repeating the analyses after removing these three individuals. Results are reported in the main text and in the Supplementary material. All statistical analyses were conducted using IBM SPSS Statistics 29.0, with statistical significance set at p < 0.05.

## 3. Results

Demographic characteristics for the participants and average performance data in the EEfRT are displayed in Table 1.

Details indicating successful manipulation of the LPS administration, that is, changes in IL-6 concentrations, in body temperature, and in sickness symptoms in both groups, can be found in Lasselin et al. (2020). To note, LPS administration, but not placebo administration, induced strong increases in IL-6 concentrations, in body temperature, and in sickness symptoms, with similar amplitude between the obese and normal-weight groups (Lasselin et al., 2020). Levels of IL-6 concentrations were clearly elevated at the time of the EEfRT (Table 1).

Details about changes in inflammatory levels in response to LPS administration between obese and normal-weight individuals in this sample were reported separately by Lasselin et al. (2020). In short, no group differences in inflammatory responses to LPS were detected (Lasselin et al., 2020).

Performance data indicate that both obese and normal-weight individuals were faster in making a choice and in completing the task in the LPS condition, compared to the placebo condition. Obese individuals were also faster in completing the task than normal-weight individuals. Completion rate was in average 97%, with a range of 74.6%−100%, and was not statistically different between groups and conditions.

### 3.1. Obesity status and chronic inflammation (Aim 1)

In the placebo condition, obese participants made less hard task choice compared to normal-weight participants when reward magnitude and probability were at their reference values (i.e., low reward and low probability) (significant main effect of obesity, Table 2, Fig. 1A). When conducting sensitivity analyses, removing the three normal-weight individuals with high proportion of high-effort tasks did not change the patterns of this result but diminished the effect (*p* = 0.055; Supplementary Table S1).

The interaction between obesity status and reward probability was not significant (Table 2, Fig. 1B). However, we found a significant interaction between obesity status and reward magnitude (significant interaction obesity*reward, Table 2). Specifically, when the reward was low, obese participants chose fewer hard tasks than normal-weight participants (<2€: *B* = −1.195, *SE* = 0.470, *p* = 0.011; 2–2.99€: *B* = −0.906, *SE* = 0.310, *p* = 0.004). As the reward increased, obese participants tended to choose more hard tasks, making their choices like those of normal-weight participants at higher reward levels (3–3.99€: *B* = −0.507, *SE* = 0.296, *p* = 0.087; ≥4€: *B* = −0.499, *SE* = 0.288, *p* = 0.083) (Fig. 1C). However, the interaction obesity*reward magnitude did not hold in sensitivity analyses and should thus be cautiously interpreted (*p* = 0.364, Supplementary Table S1).

**Fig. 2.**
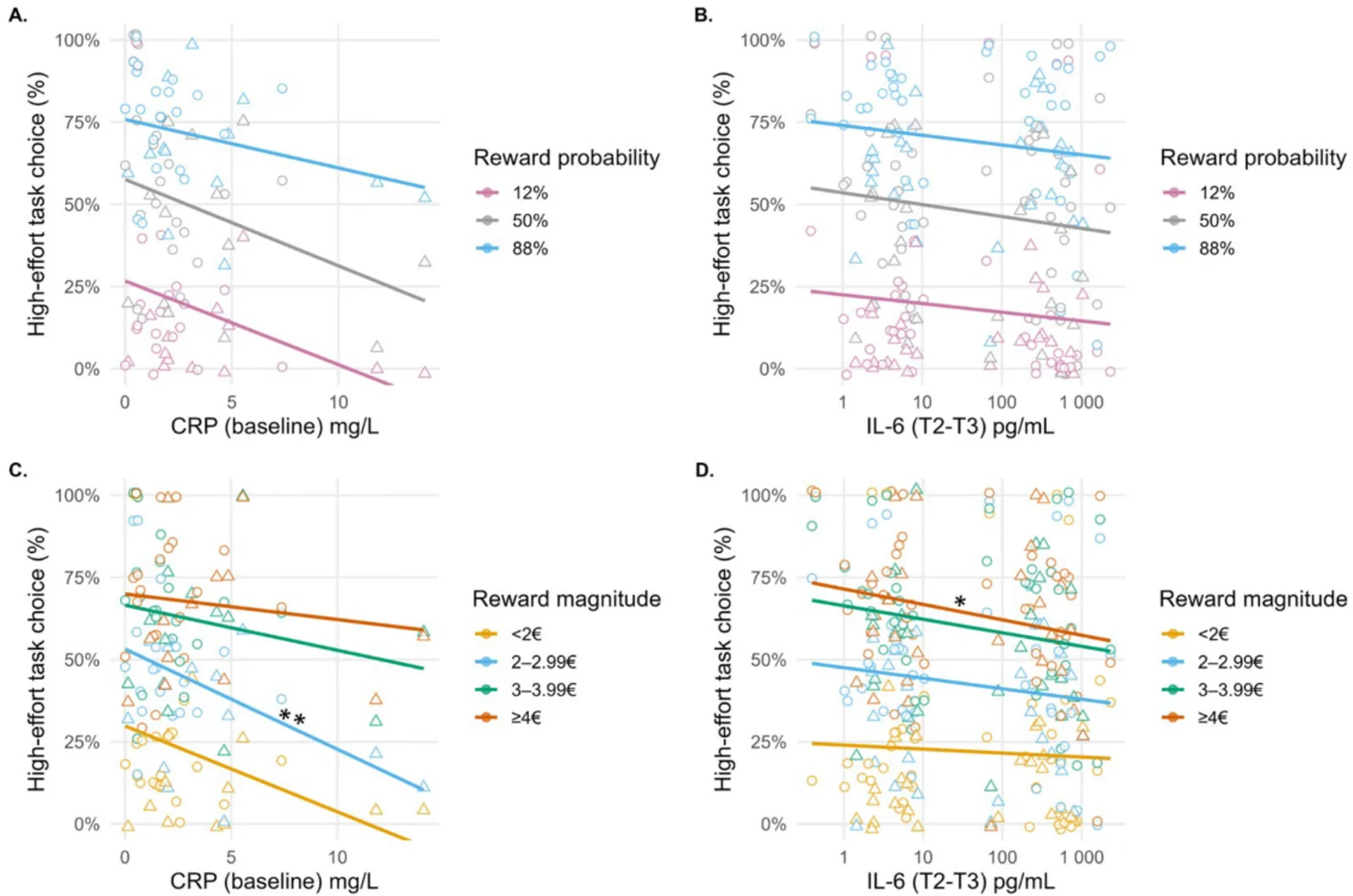
Effect of chronic inflammation (baseline CRP in placebo condition) and acute inflammation (IL-6 levels during the EEfRT in both conditions) on high effort task choice (%) for each reward probability (A-B) and each reward magnitude (C-D). Data from both groups (normal weight and obese) are combined. Individual data points represent proportion of high-effort task choices for each participant at each condition and has been jittered. Triangles represent obese individuals and circles represent normal-weight individuals. The reward magnitude (1.24–4.21 €) was divided into four categories (<2€, 2–2.99€, 3–3.99€, and ≥ 4€) for exploratory post-hoc analyses. *p < 0.05, **p < 0.01. Abbreviations: CRP=C-reactive protein, IL-6 = Interleukin-6.

Higher CRP concentrations at baseline across all volunteers predicted fewer hard-task choices when reward magnitude and probability were at their reference values (i.e., low reward and low probability) in the placebo condition (significant effect of CRP concentrations; Table 2, Fig. 2A and 2C). This effect remained consistent in direction and significant when conducting sensitivity analyses (p = 0.038; Supplementary Table S1).

We detected a significant interaction effect between baseline CRP and reward probability and between baseline CRP and reward magnitude (Table 2). Exploratory post-hoc analyses indicated a trend for higher CRP to be associated with less hard-task choice made, when reward probability was low (Fig. 2A; 12%: *B* = −0.278, *SE* = 0.146, *p* = 0.057; 50%: *B* = −0.88, *SE* = 0.055, *p* = 0.105; 88%: *B* = −0.056, *SE* = 0.031, *p* = 0.068) and when reward magnitude was low (Fig. 2C; <2€: *B* = −0.141, *SE* = 0.081, *p* = 0.081; 2–2.99€: *B* = −0.142, *SE* = 0.044, *p* = 0.001; 3–3.99€: *B* = −0.039, *SE* = 0.043, *p* = 0.357; ≥4€: *B* = −0.037, *SE* = 0.041, *p* = 0.372). However, the interactions baseline CRP*probability levels and baseline CRP*reward magnitude did not hold when conducting sensitivity analyses and should thus be cautiously interpreted (respectively, *p* = 0.238 and *p* = 0.207, Supplementary Table S1).

### 3.2. Acute inflammation (Aim 2)

No significant effect was observed in the models examining the impact of LPS condition on effort (Table 3). We also did not find a significant main effect of LPS-induced IL-6 concentrations on the overall choice of hard tasks during EEfRT (Table 3). Additionally, there was no significant interaction between IL and 6 at the time of EEfRT and reward probability (Table 3; Fig. 2B). However, a significant negative interaction was detected between IL and 6 at the time of EEfRT and reward magnitude on effort (Table 3). Specifically, higher IL-6 concentration was associated with diminished effort when the reward magnitude was high (Fig. 2D; <2€: *B* = −0.00004, *SE* = 0.0002, *p* = 0.849; 2–2.99€: *B* = −0.0002, *SE* = 0.0002, *p* = 0.361; 3–3.99€: *B* = −0.0003, *SE* = 0.0002, *p* = 0.054; ≥4€: *B* = −0.0005, *SE* = 0.0002, *p* = 0.022). This interaction persisted after conducting sensitivity analyses that excluded the three normal-weight participants with a high proportion of hard-task choices (*p* = 0.005, Supplementary Table S2).

Exploratory analyses of the effect of TNF-a concentrations and its interaction with reward magnitude and probability level were non-significant (Supplementary Table S3).

### 3.3. Interaction effects of acute inflammatory responses with obesity status and chronic inflammation (aims 3 and 4)

We did not detect a significant interaction effect between IL and 6 at the time of EEfRT and OB status on effort (Table 4, Supplementary Table S4). We also did not detect an interaction effect between IL and 6 at the time of EEfRT and baseline CRP on effort (Table 4, Supplementary Table S4).

## 4. Discussion

This randomized controlled study examined different aspects of reward-related motivation in response to immune challenge among obese individuals compared to those with normal weight, specifically in the context of higher levels of low-grade inflammation. Obese individuals, compared to normal-weight individuals, exhibited lower willingness to expend effort in order to obtain reward in the EEfRT (aim 1a). Similarly, higher baseline concentration of CRP was related to a lower reward motivation (aim 1b), and both of these effects hold with sensitivity analyses. This effect could not be explained by physical limitation since obese individuals were even faster in completing the task compared to normal-weight individuals. We observed an interaction with reward magnitude, with both obesity and higher CRP concentrations being associated with less hard-task choice when reward magnitude was low but not when it was higher; but these results did not hold the sensitivity analyses. Acute inflammation, as measured with LPS-induced interleukin-6 concentrations, also led to changes in reward motivation, in particular to a reduced effect of reward magnitude on the willingness to make an effort (aim 2), and this effect was robust to sensitivity analyses. Here as well, the effect was not explained by the physical effects of LPS, since individuals were faster in completing the task in the LPS condition compared to the placebo condition. Unexpectedly, we did not observe significant amplifying effects of obesity or low-grade inflammation on reward motivation during immune challenges. These findings were obtained in participants who were not taking medications, had no current or past history of medical or psychiatric health issues, and did not engage in behaviors associated with inflammation, such as heavy drinking or substance use, preliminarily ruling out the possibility that the results were simply due to these factors.

Our study shows that obesity and low-grade inflammation, indicated by baseline CRP, tend to have lower reward motivation. These findings align with existing research, which suggests that obesity and markers of systemic inflammation are linked to reduced reward motivation (Felger & Treadway, 2017; Mata et al., 2017). The relationships might be more complex, as differences were seen at lower reward magnitudes (for CRP and obesity status) and at lower probabilities (for CRP only), but not at higher reward magnitude or higher probability. However, these interaction results (i.e., obesity status by magnitude, baseline CRP by magnitude, and baseline CRP by reward probability) did not hold significance in sensitivity analyses removing three outliers who chose the hard task in over 95% of trials, indicating the interaction effects may lack robustness. Nevertheless, previous work using EEfRT in overweight individuals with major depression reported similar effect (Mansur et al., 2017), which might suggest a higher reward threshold for increasing effort. These interaction findings can be explained by an efficiency perspective, meaning decisions tend to favor conserving energy, and when energy expenditure is necessary, effort goes toward more valuable options. In fact, participants with higher baseline CRP catch up to those with lower CRP in the proportion of hard choices made when the potential reward is higher. Consistent with this efficiency idea, participants completed the tasks faster during the LPS condition than during the placebo condition, and obese participants generally completed tasks faster than normal-weight participants. The non-significant effects in sensitivity analyses could be due to limited statistical power from a relatively small sample size (which was due to challenges in recruiting individuals with obesity without current or past history of medical or psychiatric conditions who were also willing to participate in such experimental study). Future research should involve larger samples to better examine these nuanced relationships, specifically whether inflammation consistently reduces reward motivation or alters it in a way that promotes energy efficiency. Nevertheless and importantly, the overall effects of obesity and baseline CRP on a reduced willingness to expend effort to obtain a reward were robust in sensitivity analyses.

Notably, obesity status in this sample was characterized by low-grade inflammation (Lasselin et al., 2020). This suggests that low-grade inflammation might underlie the observed link between obesity and reward motivation. Consistent with this view, the results related to obesity and baseline CRP levels were largely similar. Although CRP is also regarded as a metabolic risk factor, in this case, the association between obesity and reward motivation was more likely driven by inflammation rather than metabolic dysregulation, since participants were screened for the latter through electrocardiogram and blood analysis. However, because obesity is also linked to factors beyond low-grade inflammation, such as stigma and discrimination (Westbury et al., 2023), these factors may have contributed as well. Additionally, although the study excluded individuals with a history of medical and psychiatric conditions, it remains unclear whether subclinical health issues influenced the associations. Future research should compare chronic inflammation with these underlying factors to confirm that low-grade inflammation associated with obesity truly connects obesity with altered reward motivation. Examining individuals with other health conditions related to inflammation could help replicate this work for the same purpose.

Greater acute inflammatory responses to immune challenge, as indicated by higher increases in IL-6 at the time of the EEfRT, was associated with a weaker effect of reward magnitude on increasing effort. This difference in willingness to expend effort became more pronounced as the reward value increased, suggesting a blunted responsivity to reward salience. Excessive acute immune activation may have reorganized the motivational state, making energy conservation and metabolic resources more salient than monetary incentives. The perceived costs or risks associated with exerting effort, such as increased fatigue, discomfort, or potential exacerbation of inflammation, might have outweighed the benefits of pursuing rewards. This finding largely aligns with the existing literature. In other studies (Draper et al., 2018; Lambregts et al., 2023), lower reward motivation was observed two hours following LPS administration. LPS administration was associated with lower neural anticipation in ventral striatum (Eisenberger et al., 2010; Moieni et al., 2019). Costi et al. (2021) reported that higher pro-inflammatory cytokine production by LPS-stimulated peripheral blood mononuclear cells was associated with reduced responses to reward anticipation within the ventral striatum and with reduced self-report anticipation of pleasure. Studies on immune challenge induced by vaccination similarly detected lower reward motivation following the intervention (Boyle et al., 2019; Harrison et al., 2016). However, several groups, including ours, detected higher reward motivation following LPS injection (Inagaki et al., 2015; Jolink et al., 2022; Lasselin et al., 2017; Muscatell et al., 2016), although these findings are not necessarily contradictory. Drawing on integrated reinforcement theories, the relationship may depend on several contextual factors, such as type of reward or physiological state, such as sleepiness (Lasselin et al., 2017) or the phase of the acute inflammatory response (early after LPS injection vs. recovery period).

Of note, associations were observed between LPS-induced IL-6 and reward motivation, whereas no effects emerged for LPS (vs. placebo) administration. These two indices of inflammation differ conceptually. Whereas analyses of IL-6 index individual differences in the magnitude of inflammatory response to LPS, the LPS condition reflects exposure to immune challenge and may capture the broader components of immune cascade. Thus, significant associations with IL-6 but not with LPS administration suggest that altered reward motivation is more closely related to individual variability in inflammatory responses rather than being exposed to immune challenge alone. This interpretation is consistent with recent findings by (Boyle et al., 2025), who similarly observed that greater LPS-induced increases in IL-6 (but not LPS condition itself, using the same dose as in the current study) predicted reduced reward motivation on the EEfRT among younger adults. In addition, null effects for LPS administration may partly reflect the use of placebo as an active comparison condition, which can engage expectancy-related process known to modulate reward motivation, thereby reducing contrasts between conditions (Scott et al., 2008).

Within the same sample, this study examined the links between acute inflammatory response, low-grade inflammation, and altered reward motivation, although the patterns differed. The findings reveal potential mechanistic differences between acute and chronic inflammatory pathways. Since the associated motivational behavioral patterns vary, it is possible that clinical implications also differ between the two processes. Future research should clarify the behavioral consequences and clinical implications of acute versus more chronic inflammatory process. This should help determine the generalizability of research findings that use different inflammatory measures. Unexpectedly, our study did not find evidence that obesity or low-grade inflammation amplifies the effect of immune challenge on reward motivation. It is unclear how long the participants have experienced low-grade inflammation. However, given that they are younger and free of health conditions, it is likely too early for the amplification effect to appear. Compensatory mechanisms present in younger, healthy individuals might have prevented such effects. It is also possible that limited statistical power constrained our ability to detect such amplification effects. Future research should replicate this study with samples that better represent the chronic low-grade inflammatory state, including older age groups and individuals with obesity in pre-morbid stages, and with larger sample sizes to ensure adequate power to detect amplification effects. When recruiting based on obesity status, related information such as prior history of obesity and duration of obesity could provide additional insight into wear-and-tear levels. Employing longitudinal designs would allow assessment of amplification effects as they relate to age and wear-and-tear status.

This study employed a randomized double-blind, placebo-controlled, crossover design, which enabled the detection of more nuanced associations between inflammation and reward motivation. Additionally, the study used multiple methods to verify the success of the manipulation. Despite its strengths, the results should be interpreted with awareness of several limitations. First, while conducting the study in a controlled laboratory setting is a strength in mitigating potential confounders, it may also limit ecological validity. Second, growing research suggests that the inflammation-reward association depends on various factors. However, these factors were not adequately accounted for (e.g., social vs. non-social rewards, prior life experiences or stress exposure, personality traits, etc. (Chat et al., 2021, 2022; Eisenberger et al., 2017; Lasselin et al., 2017)). Furthermore, although the absence of medical and psychiatric history enhances confidence in the findings by mitigating potential confounders, it may also restrict the generalizability to other populations, including obese individuals with comorbid health conditions. Third, since the immune challenge was a single dose proportional to body mass, it remains unclear whether results are specific to this particular administration. It is also unclear whether the use of reduced weight-adjusted LPS dosing (e.g., in individuals with obesity) may have introduced differences in the effective magnitude of the acute inflammatory challenge across groups, although this dosing strategy did elicit comparable inflammatory exposure across participants (Lasselin et al., 2020). Moreover, the acute inflammatory response was indexed using a limited set of circulating cytokines, which may not fully capture the complexity or temporal dynamics of the acute immune process. Future research should explore factors such as the duration of immune challenge exposure, dosage, type of challenge (e.g., vaccine, psychosocial stress-induced inflammation), and multi-marker inflammatory profile. Fourth, perceived ability to complete hard tasks was not assessed, although it may represent an alternative explanation for effort choice rather than altered reward motivation (Handke et al., 2020). Finally, the relatively small sample size, particularly the obesity group, limited statistical power for some analyses, although the study incorporated design and analytical features that are critical for improving statistical power beyond increasing samples (Lipsey, 1990; Meyvis & Van Osselaer, 2018). In addition, this limitation likely contributed to models failing to converge when sex was included as a covariate, especially given the unequal sex distribution between obesity and normal-weight groups. Given the well-documented sex differences in inflammatory responses (Fish, 2008; Klein et al., 2010) and in behavioral response to LPS (Lasselin et al., 2018), it will be important to investigate the role of gender and biological sex in future studies.

### 4.1. Conclusions

Overall, inflammatory processes, including both LPS-induced and chronic low-grade inflammation as indicated by CRP and obesity, may facilitate how effort is allocated towards reward. Chronic, low-grade inflammation was associated with reduced willingness to expend effort for reward, whereas LPS-induced inflammation was associated with dampened sensitivity to reward salience. This study provides a more nuanced understanding of how inflammatory processes may alter motivational aspects of reward, which represents an important first step toward translating this immune-reward knowledge to theoretically coherent interventions designed to prevent or treat relevant symptoms like anhedonia and deficits in reward function.

## Supplementary Material

1

## Figures and Tables

**Table 1:** Demographics and performance data in the EEfRT.

*Demographic data*	Obese (N = 14)		Normal-weight (N = 21)	

**Age – years, m (SD)**	27.6 (4.2)		24.8 (3.5)*	
**Sex – N of females, N (%)**	5 (35.7%)		13 (61.9%)	
**Weight – kg, m (SD)**	115.3 (15.9)		68.9 (8.1)	
**BMI – kg/m^2^, m (SD)**	37.0 (6.2)		22.6 (1.6)	
*Levels of inflammation*	Placebo^1^	LPS	Placebo	LPS

**Baseline CRP, mg/L (SD)**	4.4 (4.1)	5.5 (5.6)	1.9 (1.7)**	1.8 (2.7)**
**IL-6 during EEfRT, pg/mL (SD)**	4.5 (2.4)	420.8 (284.5)	4.1 (2.7)	638.8 (572.1)
*Average performance in the EEfRT*	Placebo^1^	LPS	Placebo	LPS
**Reaction time to choice – sec, m (SD)** ^2^	1.5 (0.6)	1.4 (0.5) ^#^	1.6 (0.7)	1.5 (0.6) ^#^
**Completion time – sec, m (SD)** ^2^	10.5 (6.5)	10.0 (6.4)^†^	12.3 (6.9)***	11.7 (6.7)*** ^†^
**100% successful rate – N (%)**	5 (39)	7 (50)	14 (67)	10 (48)

The significance indicates results from t-tests (χ^2^ test for sex) to assess differences between obese and normal-weight individuals in demographic data and levels of inflammation, and linear mixed models to assess differences in performance between obese and normal-weight individuals in the two conditions.

* p < 0.05

** p < 0.01

*** p < 0.001 obese vs normal-weight (main effect).

† p = 0.05

# p < 0.05

## p < 0.01 LPS vs placebo (main effect).

1 n = 13

2 no interaction effect.

Abbreviations: BMI = Body Mass Index; CRP: C-reaction protein; IL-6; Interleukin-6; EEfRT: Effort Expenditure for Reward Task.

**Table 2:** Effect of obesity and low-grade inflammation on EEfRT outcomes in the placebo condition.

	Obesity				Baseline CRP concentrations			
	*B*	*SE*	95% CI	*p*	*B*	*SE*	95% CI	*p*

**Reward magnitude**	1.03	0.18	0.68; 1.38	0<.001	0.86	0.19	0.49; 1.24	0<.001
**Probability level**	3.51	0.57	2.40; 4.63	0<.001	2.98	0.62	1.78; 4.19	0<.001
**Obesity (vs normal-weight) / Baseline CRP concentrations**	−3.11	1.16	−5.37; −0.84	0.007	−0.80	0.31	−1.41; −0.19	0.010
Obesity/CRP*reward magnitude	0.42	0.21	0.02; 0.83	0.041	0.12	0.06	0.01; 0.24	0.040
Obesity/CRP*probability level	1.260	0.75	−0.21; 2.73	0.094	0.38	0.19	0.01; 0.74	0.045
***Covariates***								
**EEfRT Trial**	−0.02	0.003	−0.02; −0.01	0<.001	−0.02	0.003	−0.02; −0.01	0<.001
**Study session**	0.54	0.42	−0.29; 1.37	0.203	0.45	0.43	−0.39; 1.30	0.290
**Age**	0.05	0.06	−0.08; 0.17	0.464	0.02	0.06	−0.09; 0.14	0.698
**Woman (vs man)**	– -	– -	– -	– -	−0.08	0.41	−0.88; 0.73	0.851

Generalized estimating equation (GEE) using exchangeable variance structure on high-effort task choice (no/yes) as dependent variable. The model with obesity did not converge when sex was added as covariate.

Note that the interactions obesity/CRP*reward magnitude and CRP*probability level were not significant in sensitivity analyses removing three individuals with extremely high percentage of hard-task choices (>95%) and should thus be cautiously interpreted.

*Abbreviations:* EEfRT: Effort Expenditure for Reward Task, CRP: C-reactive protein.

**Table 3:** Effect of acute inflammation on EEfRT outcomes.

	LPS				IL-6 concentrations			
	*B*	*SE*	95% CI	*p*	*B*	*SE*	95% CI	*p*

**Reward magnitude**	1.11	0.14	0.84; 1.38	0<.001	1.10	0.13	0.84; 1.37	0<.001
**Probability level**	3.73	0.46	2.83; 4.62	0<.001	3.65	0.48	2.70; 4.60	0<.001
**LPS (vs placebo) / IL-6 concentrations**	0.37	0.47	−0.55; 1.30	0.426	0.001	0.0007	−0.001; 0.002	0.479
LPS/IL-6*reward magnitude	−0.16	0.11	−0.37; 0.05	0.129	−0.0002	0.0001	−0.001; −0.00002	0.033
LPS/IL-6*probability level	−0.18	0.34	−0.83; 0.48	0.602	−0.00003	0.0008	−0.002; 0.002	0.962
***Covariates***								
**EEfRT Trial**	−0.01	0.003	−0.02; −0.01	0<.001	−0.01	0.003	−0.02; −0.007	0<.001
**Study session**	−0.07	0.09	−0.24; 0.10	0.435	−0.08	0.08	−0.23; 0.08	0.350
**Age**	−0.06	0.08	−0.21; 0.09	0.433	−0.06	0.08	−0.21; 0.09	0.454
**Woman (vs man)**	– -	– -	– -	– -	– -	– -	– -	– -

Generalized estimating equation (GEE) using exchangeable variance structure on high-effort task choice (no/yes) as dependent variable. Both models did not converge when sex was added as covariate.

*Abbreviations:* EEfRT: Effort Expenditure for Reward Task, LPS: lipopolysaccharide, IL-6: interleukin-6.

**Table 4:** Interaction between low-grade inflammation and acute inflammation on EEfRT outcomes.

	Obesity * IL-6 concentrations				Baseline CRP concentrations * IL-6 concentrations			
	*B*	*SE*	95% CI	*p*	*B*	*SE*	95% CI	*p*

**Reward magnitude**	0.83	0.10	0.63; 1.03	0<.001	0.78	0.09	0.60; 0.97	0<.001
**Obesity (vs normal-weight) / Baseline CRP concentrations**	−1.46	0.61	−2.66; −0.26	0.017	−0.17	0.08	−0.32; −0.02	0.031
Obesity/CRP*reward magnitude	0.22	0.14	−0.06; 0.50	0.117	0.04	0.02	−0.002; 0.09	0.064
**IL-6 concentrations**	0.0003	0.0002	−0.0001; 0.001	0.155	0.0001	0.0003	−0.0005; 0.001	0.669
IL-6*reward magnitude	−0.0002	0.0001	0.0004; 0.00004	0.017	−0,0001	0.0001	−0.0003; 0.0001	0.157
Obesity (vs normal-weight) / Baseline CRP concentrations*IL-6 concentrations	0.001	0.0008	−0.001; 0.002	0.397	0.0002	0.0001	−0.00004; 0.0004	0.114
Obesity/CRP*IL-6 concentrations*reward magnitude	−0.0002	0.0003	−0.001; 0.0004	0.595	−0,00005	0.00004	−0.0001; 0.00002	0.173
***Covariates***								
**EEfRT Trial**	−0.01	0.002	−0.01; −0.006	0<.001	−0.01	0.002	−0.01; −0.006	0<.001
**Study session**	−0.06	0.07	−0.19; 0.07	0.353	−0.08	0.07	−0.23; 0.06	0.260
**Age**	0.04	0.05	−0.06; 0.15	0.413	0.004	0.05	−0.10; 0.11	0.945
**Woman (vs man)**	– -	– -	– -	– -	−0.42	0.33	−1.06; 0.22	0.199

Generalized estimating equation (GEE) using exchangeable variance structure on high-effort task choice (no/yes) as dependent variable. Model with obesity did not converge when sex was added as covariate.

*Abbreviations:* EEfRT: Effort Expenditure for Reward Task, CRP: C-reactive protein, LPS: lipopolysaccharide, IL-6: interleukin-6.

## Data Availability

The authors do not have permission to share data.

## Appendix A. Supplementary data

Supplementary data to this article can be found online at https://doi.org/10.1016/j.bbi.2026.106548.

## References

[R1] Aguliar-Valles A, Kim J, Jung S, Woodside B, Luheshi GN, 2014. Role of brain transmigrating neutrophils in depression-like behavior during systemic infection. Mol. Psychiatry 19 (5), 599–606. 10.1038/mp.2013.137. 24126927

[R2] Banks WA, Kastin AJ, Broadwell RD, 1995. Passage of cytokines across the blood-brain barrier. Neuroimmunomodulation 2 (4), 241–248. 10.1159/000097202. 8963753

[R3] Bower JE, Kuhlman KR, 2023. Psychoneuroimmunology: an Introduction to Immune-to-Brain Communication and its Implications for Clinical Psychology. Annu. Rev. Clin. Psychol. 19, 331–359. 10.1146/annurev-clinpsy-080621-045153. 36791765

[R4] Boyle CC, Cho JH, Eisenberger NI, Olmstead R, Sadeghi N, Castillo D, Irwin MR, 2025. Inflammation-induced depressed mood and reward responsivity as a function of age in female adults: a randomized controlled trial of endotoxin. Transl. Psychiatry 16 (1), 6. 10.1038/s41398-025-03752-2. 41298372 PMC12783272

[R5] Boyle CC, Kuhlman KR, Dooley LN, Haydon MD, Robles TF, Ang Y-S, Pizzagalli DA, Bower JE, 2019. Inflammation and dimensions of reward processing following exposure to the influenza vaccine. Psychoneuroendocrinology 102, 16–23. 10.1016/j.psyneuen.2018.11.024. 30496908 PMC6420390

[R6] Chat I-K-Y, Gepty AA, Kautz M, Giollabhui NM, Adogli ZV, Coe CL, Abramson LY, Olino TM, Alloy LB, 2022. Residence in High-Crime Neighborhoods Moderates the Association between Interleukin 6 and Social and Nonsocial Reward Brain responses. Biological Psychiatry Global Open Science, Exposome: Understanding Environmental Impacts on Brain Development and Risk for Psychopathology 2 (3), 273–282. 10.1016/j.bpsgos.2022.04.006. PMC9306340 35873737

[R7] Chat I-K-Y, Nusslock R, Moriarity DP, Bart CP, Mac Giollabhui N, Damme KSF, Carroll AL, Miller GE, Alloy LB, 2021. Goal-striving tendencies moderate the relationship between reward-related brain function and peripheral inflammation. Brain Behav. Immun. 94, 60–70. 10.1016/j.bbi.2021.03.006. 33705866 PMC8075112

[R8] Costi S, Morris LS, Collins A, Fernandez NF, Patel M, Xie H, Kim-Schulze S, Stern ER, Collins KA, Cathomas F, Parides MK, Whitton AE, Pizzagalli DA, Russo SJ, Murrough JW, 2021. Peripheral immune cell reactivity and neural response to reward in patients with depression and anhedonia. Transl. Psychiatry 11 (1), 565. 34741019 10.1038/s41398-021-01668-1 PMC8571388

[R9] Cox AJ, West NP, Cripps AW, 2015. Obesity, inflammation, and the gut microbiota. The Lancet Diabetes & Endocrinology 3 (3), 207–215. 10.1016/S2213-8587(14)70134–2. 25066177

[R10] Dantzer R, 2023. Evolutionary Aspects of Infections: Inflammation and Sickness Behaviors. Curr. Top. Behav. Neurosci. 61, 1–14. 10.1007/7854_2022_363. 35505054 PMC10071523

[R11] Dantzer R, Kelley KW, 2007. Twenty years of research on cytokine-induced sickness behavior. Brain Behav. Immun. 21 (2), 153–160. 10.1016/j.bbi.2006.09.006. 17088043 PMC1850954

[R12] Dantzer R, O’Connor JC, Freund GG, Johnson RW, Kelley KW, 2008. From inflammation to sickness and depression: when the immune system subjugates the brain. Nat. Rev. Neurosci. 9 (1), 46–56. 10.1038/nrn2297. 18073775 PMC2919277

[R13] De Marco R, Barritt AW, Cercignani M, Cabbai G, Colasanti A, Harrison NA, 2023. Inflammation-induced reorientation of reward versus punishment sensitivity is attenuated by minocycline. Brain Behav. Immun. 111, 320–327. 10.1016/j.bbi.2023.04.010. 37105388

[R14] Dooley LN, Kuhlman KR, Robles TF, Eisenberger NI, Craske MG, Bower JE, 2018. The role of inflammation in core features of depression: Insights from paradigms using exogenously-induced inflammation. Neurosci. Biobehav. Rev. 94, 219–237. 10.1016/j.neubiorev.2018.09.006. 30201219 PMC6192535

[R15] Draper A, Koch RM, van der Meer JW, Apps AJ, M., Pickkers P, Husain M, van der Schaaf ME, 2018. Effort but not Reward Sensitivity is Altered by Acute Sickness Induced by Experimental Endotoxemia in Humans. Article 5 Neuropsychopharmacology 43 (5). 10.1038/npp.2017.231. PMC5854801 28948979

[R16] Eisenberger NI, Berkman ET, Inagaki TK, Rameson LT, Mashal NM, Irwin MR, 2010. Inflammation-induced anhedonia: Endotoxin reduces ventral striatum responses to reward. Biol. Psychiatry 68 (8), 748–754. 10.1016/j.biopsych.2010.06.010. 20719303 PMC3025604

[R17] Eisenberger NI, Moieni M, Inagaki TK, Muscatell KA, Irwin MR, 2017. In Sickness and in Health: the Co-Regulation of Inflammation and Social Behavior. Neuropsychopharmacology: Official Publication of the American College of.Neuropsychopharmacology 42 (1), 242–253. 10.1038/npp.2016.141. 27480575 PMC5143485

[R18] Engler H, Brendt P, Wischermann J, Wegner A, Rӧhling R, Schoemberg T, Meyer U, Gold R, Peters J, Benson S, 2017. Selective increase of cerebrospinal fluid IL-6 during experimental systemic inflammation in humans: Association with depressive symptoms. Mol. Psychiatry 22 (10), 1448–1454. 28138158 10.1038/mp.2016.264

[R19] Felger JC, Li Z, Haroon E, Woolwine BJ, Jung MY, Hu X, Miller AH, 2016. Inflammation is associated with decreased functional connectivity within corticostriatal reward circuitry in depression. Mol. Psychiatry 21 (10), 1358–1365. 10.1038/mp.2015.168. 26552591 PMC4862934

[R20] Felger JC, Treadway MT, 2017. Inflammation Effects on Motivation and Motor activity: Role of Dopamine. Neuropsychopharmacology: Official Publication of the American College of. Neuropsychopharmacology 42 (1), 216–241. 10.1038/npp.2016.143. 27480574 PMC5143486

[R21] Fish EN, 2008. The X-files in immunity: Sex-based differences predispose immune responses. Nat. Rev. Immunol. 8 (9), 737–744. 10.1038/nri2394. 18728636 PMC7097214

[R22] Gregor MF, Hotamisligil GS, 2011. Inflammatory Mechanisms in Obesity. Annu. Rev. Immunol. 29(Volume 29, 415–445. 10.1146/annurev-immunol-031210-101322. 21219177

[R23] Handke A, Axelsson J, Benson S, Boy K, Weskamp V, Hasenberg T, Remy M, Hebebrand J, Fӧcker M, Brinkhoff A, Unteroberdӧrster M, Engler H, Schedlowski M, Lasselin J, 2020. Acute inflammation and psychomotor slowing: Experimental assessment using lipopolysaccharide administration in healthy humans. Brain, Behavior, & Immunity - Health 8, 100130. 10.1016/j.bbih.2020.100130. PMC8474655 34589881

[R24] Hansson LS, Tognetti A, Sigurjónsson P, Brück E, Wåhlén K, Jensen K, Olsson MJ, Toll John R, Wilhelms DB, Lekander M, Lasselin J, 2024. Perception of unfamiliar caregivers during sickness – using the new Caregiver perception Task (CgPT) during experimental endotoxemia. Brain Behav. Immun. 119, 741–749. 10.1016/j.bbi.2024.04.031. 38670241

[R25] Harrison NA, Voon V, Cercignani M, Cooper EA, Pessiglione M, Critchley HD, 2016. A Neurocomputational Account of how Inflammation Enhances Sensitivity to Punishments Versus Rewards. Biol. Psychiatry 80 (1), 73–81. 10.1016/j.biopsych.2015.07.018. 26359113 PMC4918729

[R26] Huang X, Hussain B, Chang J, 2021. Peripheral inflammation and blood–brain barrier disruption: Effects and mechanisms. CNS Neurosci. Ther. 27 (1), 36–47. 10.1111/cns.13569. 33381913 PMC7804893

[R27] Inagaki TK, Muscatell KA, Irwin MR, Moieni M, Dutcher JM, Jevtic I, Breen EC, Eisenberger NI, 2015. The role of the ventral striatum in inflammatory-induced approach toward support figures. Brain Behav. Immun. 44, 247–252. 10.1016/j.bbi.2014.10.006. 25459101 PMC4275369

[R28] Jolink TA, Fendinger NJ, Alvarez GM, Feldman MJ, Gaudier-Diaz MM, Muscatell KA, 2022. Inflammatory reactivity to the influenza vaccine is associated with changes in automatic social behavior. Brain Behav. Immun. 99, 339–349. 34748895 10.1016/j.bbi.2021.10.019 PMC9041378

[R29] Kanneganti T-D, Dixit VD, 2012. Immunological complications of obesity. Nat. Immunol. 13 (8), 707–712. 10.1038/ni.2343. 22814340

[R30] Kemna E, Pickkers P, Nemeth E, van der Hoeven H, Swinkels D, 2005. Time-course analysis of hepcidin, serum iron, and plasma cytokine levels in humans injected with LPS. Blood 106 (5), 1864–1866. 10.1182/blood-2005-03-1159. 15886319

[R31] Klein SL, Jedlicka A, Pekosz A, 2010. The Xs and Y of immune responses to viral vaccines. Lancet Infect. Dis. 10 (5), 338–349. 10.1016/S1473-3099(10)70049-9. 20417416 PMC6467501

[R32] Kushner I, Rzewnicki D, Samols D, 2006. What does Minor Elevation of C-Reactive Protein Signify? Am. J. Med. 119 (2), 166.e17–166.e28. 10.1016/j.amjmed.2005.06.057. 16443421

[R33] Lacourt TE, Vichaya EG, Escalante C, Manzullo EF, Gunn B, Hess KR, Heijnen CJ, Dantzer R, 2018. An effort expenditure perspective on cancer-related fatigue. Psychoneuroendocrinology 96, 109–117. 29929087 10.1016/j.psyneuen.2018.06.009 PMC6131045

[R34] Lambregts BIHM, Vassena E, Jansen A, Stremmelaar DE, Pickkers P, Kox M, Aarts E, van der Schaaf ME, 2023. Fatigue during acute systemic inflammation is associated with reduced mental effort expenditure while task accuracy is preserved. Brain Behav. Immun. 112, 235–245. 10.1016/j.bbi.2023.05.013. 37257522

[R35] Lasselin J, Benson S, Hebebrand J, Boy K, Weskamp V, Handke A, Hasenberg T, Remy M, Fӧcker M, Unteroberdӧrster M, Brinkhoff A, Engler H, Schedlowski M, 2020. Immunological and behavioral responses to in vivo lipopolysaccharide administration in young and healthy obese and normal-weight humans. Brain Behav. Immun. 88, 283–293. 10.1016/j.bbi.2020.05.071. 32485294

[R36] Lasselin J, Lekander M, Axelsson J, Karshikoff B, 2018. Sex differences in how inflammation affects behavior: what we can learn from experimental inflammatory models in humans. Frontiers in Neuroendocrinology, Drug Development for Brain Disorders: Why Sex Matters 50, 91–106. 10.1016/j.yfrne.2018.06.005. 29935190

[R37] Lasselin J, Lekander M, Benson S, Schedlowski M, Engler H, 2021. Sick for science: Experimental endotoxemia as a translational tool to develop and test new therapies for inflammation-associated depression. Mol. Psychiatry 26 (8), 3672–3683. 10.1038/s41380-020-00869-2. 32873895 PMC8550942

[R38] Lasselin J, Magne E, Beau C, Ledaguenel P, Dexpert S, Aubert A, Layé S, Capuron L, 2014. Adipose inflammation in obesity: Relationship with circulating levels of inflammatory markers and association with surgery-induced weight loss. J. Clin. Endocrinol. Metabol. 99 (1), E53–E61. 10.1210/jc.2013-2673 24243638

[R39] Lasselin J, Treadway MT, Lacourt TE, Soop A, Olsson MJ, Karshikoff B, Paues-Gӧranson S, Axelsson J, Dantzer R, Lekander M, 2017. Lipopolysaccharide Alters Motivated Behavior in a Monetary Reward Task: A Randomized Trial 42, 801–810. 10.1038/npp.2016.191 PMC5312062 27620550

[R40] Lawrence CB, Brough D, Knight EM, 2012. Obese mice exhibit an altered behavioural and inflammatory response to lipopolysaccharide. Dis. Model. Mech. 5 (5), 649–659. 10.1242/dmm.009068. 22328591 PMC3424462

[R41] Lemmens HJM, Bernstein DP, Brodsky JB, 2006. Estimating Blood volume in Obese and Morbidly Obese patients. Obes. Surg. 16 (6), 773–776. 10.1381/096089206777346673. 16756741

[R42] Lipsey MW, 1990. Design Sensitivity: Statistical Power for Experimental Research. SAGE.

[R43] Maes M, 1995. Evidence for an immune response in major depression: a review and hypothesis. Prog. Neuropsychopharmacol. Biol. Psychiatry 19 (1), 11–38. 10.1016/0278-5846(94)00101-M. 7708925

[R44] Mata F, Treadway M, Kwok A, Truby H, Yücel M, Stout JC, Verdejo-Garcia A, 2017. Reduced Willingness to Expend Effort for Reward in Obesity: link to Adherence to a 3-month Weight loss intervention. Obesity 25 (10), 1676–1681. 10.1002/oby.21948. 28782916

[R45] Meyvis T, Van Osselaer SM, 2018. Increasing the power of your study by increasing the effect size. J. Consum. Res. 44 (5), 1157–1173.

[R46] Miller AH, Raison CL, 2016. The role of inflammation in depression: from evolutionary imperative to modern treatment target. Nat. Rev. Immunol. 16 (1), 22–34. 10.1038/nri.2015.5. 26711676 PMC5542678

[R47] Moieni M, Tan KM, Inagaki TK, Muscatell KA, Dutcher JM, Jevtic I, Breen EC, Irwin MR, Eisenberger NI, 2019. Sex differences in the Relationship between Inflammation and Reward Sensitivity: a Randomized Controlled Trial of Endotoxin. Biol. Psychiatry: Cognit. Neurosci. Neuroimaging 4 (7), 619–626. 10.1016/j.bpsc.2019.03.010. PMC6612452 31103547

[R48] Muscatell KA, Moieni M, Inagaki TK, Dutcher JM, Jevtic I, Breen EC, Irwin MR, Eisenberger NI, 2016. Exposure to an inflammatory challenge enhances neural sensitivity to negative and positive social feedback. Brain Behav. Immun. 57, 21–29. 27032568 10.1016/j.bbi.2016.03.022 PMC5011017

[R49] Nusslock R, Alloy LB, Brody GH, Miller GE, 2024. Annual Research Review: Neuroimmune network model of depression: a developmental perspective. J. Child Psychol. Psychiatry 65 (4), 538–567. 10.1111/jcpp.13961. 38426610 PMC11090270

[R50] Park HS, Park JY, Yu R, 2005. Relationship of obesity and visceral adiposity with serum concentrations of CRP, TNF-α and IL-6. Diabetes Res. Clin. Pract. 69 (1), 29–35. 10.1016/j.diabres.2004.11.007. 15955385

[R51] Pizzagalli DA, 2022. Toward a Better Understanding of the Mechanisms and Pathophysiology of Anhedonia: are we ready for translation? Am. J. Psychiatry 179 (7), 458–469. 10.1176/appi.ajp.20220423. 35775159 PMC9308971

[R52] Pohl J, Sheppard M, Luheshi GN, Woodside B, 2014. Diet-induced weight gain produces a graded increase in behavioral responses to an acute immune challenge. Brain Behav. Immun. 35, 43–50. 10.1016/j.bbi.2013.09.002. 24026015

[R53] Savitz J, Figueroa-Hall LK, Teague TK, Yeh H-W, Zheng H, Kuplicki R, Burrows K, El-Sabbagh N, Thomas M, Ewers I, Cha Y-H, Guinjoan S, Khalsa SS, Paulus MP, Irwin MR, 2025. Systemic Inflammation and Anhedonic responses to an Inflammatory Challenge in adults with Major Depressive Disorder: a Randomized Controlled Trial. Am. J. Psychiatry 182 (6), 560–568. 10.1176/appi.ajp.20240142. 40264292 PMC12997232

[R54] Scott DJ, Stohler CS, Egnatuk CM, Wang H, Koeppe RA, Zubieta J-K, 2008. Placebo and Nocebo Effects are Defined by Opposite Opioid and Dopaminergic responses. Arch. Gen. Psychiatry 65 (2), 220–231. 10.1001/archgenpsychiatry.2007.34. 18250260

[R55] Stunkard AJ, Faith MS, Allison KC, 2003. Depression and obesity. Biol. Psychiatry 54 (3), 330–337. 12893108 10.1016/s0006-3223(03)00608-5

[R56] Treadway MT, Buckholtz JW, Schwartzman AN, Lambert WE, Zald DH, 2009. Worth the ‘EEfRT’? the Effort Expenditure for Rewards Task as an Objective measure of Motivation and Anhedonia. PLoS One 4 (8), e6598. 19672310 10.1371/journal.pone.0006598 PMC2720457

[R57] Treadway MT, Cooper JA, Miller AH, 2019. Can’t or Won’t? Immunometabolic Constraints on Dopaminergic Drive. Trends Cogn. Sci. 23 (5), 435–448. 10.1016/j.tics.2019.03.003. 30948204 PMC6839942

[R58] Treadway MT, Zald DH, 2011. Reconsidering anhedonia in depression: Lessons from translational neuroscience. Neurosci. Biobehav. Rev. 35 (3), 537–555. 10.1016/j.neubiorev.2010.06.006. 20603146 PMC3005986

[R59] Vichaya EG, Dantzer R, 2018. Inflammation-induced motivational changes: Perspective gained by evaluating positive and negative valence systems. Current Opinion in Behavioral Sciences, Apathy and Motivation 22, 90–95. 10.1016/j.cobeha.2018.01.008. PMC5987547 29888301

[R60] Westbury S, Oyebode O, van Rens T, Barber TM, 2023. Obesity Stigma: Causes, Consequences, and potential Solutions. Curr. Obes. Rep. 12 (1), 10–23. 10.1007/s13679-023-00495-3. 36781624 PMC9985585

