# Supplementary material for: Obesity, low-grade inflammation, and inflammatory response to immune challenge modulate willingness to expend effort for reward
Source: Brain Behav Immun. Author manuscript; Available in PMC 2026 Aug 28. (PMC13523163; doi:10.1016/j.bbi.2026.106548)
Supplement: Supplementary file 1 [file NIHMS2196906-supplement-1.docx]

**Supplementary Table S1. Effect of obesity and low-grade inflammation on EEfRT outcomes in the placebo condition – excluding three individuals with high proportion of high-effort tasks**

|  | **Obesity** | | | |  | **Baseline CRP concentrations** | | | |
| --- | --- | --- | --- | --- | --- | --- | --- | --- | --- |
|  | ***B*** | ***SE*** | **95% CI** | ***p*** |  | ***B*** | ***SE*** | **95% CI** | ***p*** |
| **Reward magnitude** | 1.28 | 0.21 | 0.87; 1.69 | <.001 |  | 1.22 | 0.17 | 0.88; 1.55 | <.001 |
| **Probability level** | 4.27 | 0.67 | 2.96; 5.58 | <.001 |  | 4.09 | 0.55 | 3.01; 5.18 | <.001 |
| **Obesity (vs normal-weight) / Baseline CRP concentrations** | -1.91 | 0.99 | -3.85; 0.04 | .055 |  | -0.33 | 0.16 | -0.65; -0.02 | .038 |
| **Obesity/CRP*reward magnitude** | 0.20 | 0.22 | -0.23; 0.63 | .364 |  | 0.05 | 0.04 | -0.03; 0.12 | .207 |
| **Obesity/CRP*probability level** | 0.56 | 0.80 | -1.01; 2.14 | .484 |  | 0.13 | 0.11 | -0.09; 0.34 | .238 |
| ***Covariates*** |  |  |  |  |  |  |  |  |  |
| **EEfRT Trial** | -0.02 | 0.003 | -0.02; -0.01 | <.001 |  | -0.02 | 0.003 | -0.02; -0.01 | <.001 |
| **Study session** | 0.35 | 0.32 | -0.28; 0.97 | .278 |  | 0.06 | 0.35 | -0.62; 0.75 | .862 |
| **Age** | 0.01 | 0.05 | -0.08; 0.11 | .783 |  | 0.01 | 0.05 | -0.09; 0.11 | .818 |
| **Woman (vs man)** | -0.39 | 0.36 | -1.09; 0.31 | .273 |  | 0.44 | 0.39 | -0.32; 1.21 | .257 |

Generalized estimating equation (GEE) using exchangeable variance structure on high-effort task choice (no/yes) as dependent variable. *Abbreviations:* EEfRT: Effort Expenditure for Reward Task, CRP: C-reactive protein.

**Supplementary Table S2. Effect of acute inflammation on EEfRT outcomes – excluding three individuals with high proportion of high-effort tasks**

|  | **LPS** | | | |  | **IL-6 concentrations** | | | |
| --- | --- | --- | --- | --- | --- | --- | --- | --- | --- |
|  | ***B*** | ***SE*** | **95% CI** | ***p*** |  | ***B*** | ***SE*** | **95% CI** | ***p*** |
| **Reward magnitude** | 1.32 | 0.12 | 1.08; 1.56 | <.001 |  | 1.32 | 0.11 | 1.10; 1.54 | <.001 |
| **Probability level** | 4.37 | 0.42 | 3.54; 5.19 | <.001 |  | 4.32 | 0.44 | 3.46; 5.18 | <.001 |
| **LPS (vs placebo) / IL-6 concentrations** | 0.51 | 0.59 | -0.65; 1.67 | .389 |  | 0.001 | 0.0008 | -0.001; 0.002 | .237 |
| **LPS/IL-6*reward magnitude** | -0.20 | 0.13 | -0.46; 0.06 | .131 |  | −0.0004 | 0.0001 | -0.0006; -0.0001 | .005 |
| **LPS/IL-6*probability level** | -0.25 | 0.42 | -1.07; 0.57 | .553 |  | −0.0003 | 0.0009 | -0.002; 0.001 | .739 |
| ***Covariates*** |  |  |  |  |  |  |  |  |  |
| **EEfRT Trial** | -0.02 | 0.002 | -0.02; -0.01 | <.001 |  | -0.02 | 0.002 | -0.02; -0.01 | <.001 |
| **Study session** | -0.06 | 0.10 | -0.25; 0.13 | .531 |  | -0.07 | 0.09 | -0.24; 0.11 | .438 |
| **Age** | -0.04 | 0.06 | -0.15; 0.07 | .428 |  | -0.04 | 0.06 | -0.15; 0.07 | .470 |
| **Woman (vs man)** | -0.02 | 0.47 | -0.95; 0.91 | .963 |  | 0.09 | 0.48 | -0.85; 1.02 | .854 |

Generalized estimating equation (GEE) using exchangeable variance structure on high-effort task choice (no/yes) as dependent variable. *Abbreviations:* EEfRT: Effort Expenditure for Reward Task, LPS: lipopolysaccharide, IL-6: interleukin-6.

**Supplementary Table S3. Effect of TNF-α on EEfRT outcomes**

|  | **TNF-α concentrations (2-3h post-LPS)** | | | |  | **TNF-α concentrations (1-2h post-LPS)** | | | |
| --- | --- | --- | --- | --- | --- | --- | --- | --- | --- |
|  | ***B*** | ***SE*** | **95% CI** | ***p*** |  | ***B*** | ***SE*** | **95% CI** | ***p*** |
| **Reward magnitude** | 1.11 | 0.14 | 0.82; 1.39 | <.001 |  | 1.07 | 0.15 | 0.78; 1.37 | <.001 |
| **Probability level** | 3.83 | 0.48 | 2.89; 4.76 | <.001 |  | 3.82 | 0.49 | 2.86; 4.78 | <.001 |
| **TNF-α concentrations** | 0.002 | 0.003 | -0.004; 0.008 | .474 |  | 0.0001 | 0.003 | -0.005; 0.005 | .958 |
| **TNF-α*reward magnitude** | -0.001 | 0.0007 | -0.002; 0.0005 | .204 |  | −0.0003 | 0.0006 | -0.001; -0.001 | .660 |
| **TNF-α*probability level** | -0.002 | 0.002 | -0.007; 0.002 | .322 |  | −0.001 | 0.002 | -0.005; 0.003 | .492 |
| ***Covariates*** |  |  |  |  |  |  |  |  |  |
| **EEfRT Trial** | -0.01 | 0.003 | -0.02; -0.01 | <.001 |  | -0.12 | 0.003 | -0.02; -0.01 | <.001 |
| **Study session** | -0.02 | 0.07 | -0.16; 0.13 | .808 |  | -0.01 | 0.09 | -0.19; 0.18 | .933 |
| **Age** | -0.07 | 0.08 | -0.23; 0.08 | .365 |  | -0.13 | 0.09 | -0.30; 0.05 | .156 |
| **Woman (vs man)** | -- | -- | -- | -- |  | -- | -- | -- | -- |

Generalized estimating equation (GEE) using exchangeable variance structure on high-effort task choice (no/yes) as dependent variable. Similar effects were observed when excluding the three individuals with high proportion of high-effort tasks. *Abbreviations:* EEfRT: Effort Expenditure for Reward Task, LPS: lipopolysaccharide, IL-6: interleukin-6.

**Supplementary Table S4. Interaction between low-grade inflammation and acute inflammation on EEfRT outcomes – excluding three individuals with high proportion of high-effort tasks**

|  | **Obesity * IL-6 concentrations** | | | |  | **Baseline CRP concentrations * IL-6 concentrations** | | | |
| --- | --- | --- | --- | --- | --- | --- | --- | --- | --- |
|  | ***B*** | ***SE*** | **95% CI** | ***p*** |  | ***B*** | ***SE*** | **95% CI** | ***p*** |
| **Reward magnitude** | 0.99 | 0.08 | 0.83; 1.16 | <.001 |  | 0.97 | 0.07 | 0.83; 1.11 | <.001 |
| **Obesity (vs normal-weight) / Baseline CRP concentrations** | -0.85 | 0.42 | -1.68; -0.02 | .046 |  | -0.08 | 0.06 | -0.19; 0.03 | .174 |
| **Obesity/CRP*reward magnitude** | 0.09 | 0.13 | -0.17; 0.34 | .501 |  | 0.02 | 0.02 | -0.02; 0.06 | .382 |
| **IL-6 concentrations** | 0.0005 | 0.0003 | −0.000003; 0.001 | .051 |  | 0.0004 | 0.0003 | -0.0002; 0.001 | .231 |
| **IL-6*reward magnitude** | −0.0003 | 0.00008 | -0.0004; -0.0001 | <.001 |  | −0.0002 | 0.00009 | -0.0004; -0.00004 | .018 |
| **Obesity (vs normal-weight) / Baseline CRP concentrations*IL-6 concentrations** | 0.0004 | 0.0008 | -0.001; 0.002 | .618 |  | 0.00009 | 0.00009 | -0.00009; 0.0003 | .316 |
| **Obesity/CRP*IL-6 concentrations*reward magnitude** | −0.00008 | 0.0003 | -0.001; 0.0005 | .794 |  | −0.00003 | 0.00003 | −0.00009; 0.00004 | .396 |
| ***Covariates*** |  |  |  |  |  |  |  |  |  |
| **EEfRT Trial** | -0.01 | 0.002 | -0.01; -0.008 | <.001 |  | -0.01 | 0.002 | -0.01; -0.008 | <.001 |
| **Study session** | -0.06 | 0.07 | -0.20; 0.08 | .408 |  | -0.07 | 0.08 | -0.22; 0.08 | .349 |
| **Age** | 0.02 | 0.04 | -0.06; 0.11 | .574 |  | 0.007 | 0.04 | -0.07; 0.09 | .860 |
| **Woman (vs man)** | -0.28 | 0.35 | -0.97; 0.40 | .414 |  | -0.04 | 0.30 | -0.63; 0.54 | .883 |

Generalized estimating equation (GEE) using exchangeable variance structure on high-effort task choice (no/yes) as dependent variable. *Abbreviations:* EEfRT: Effort Expenditure for Reward Task, CRP: C-reactive protein, LPS: lipopolysaccharide, IL-6: interleukin-6.
